# The Use of Digital Neurocognitive Assessments to Assess Traumatic Brain Injury and Dementia in Older Trauma Patients: An Emergency Department Feasibility Study †

**DOI:** 10.3390/diagnostics16030400

**Published:** 2026-01-27

**Authors:** Justin Weppner, Justin Gray, Damon Kuehl, Danielle Sandsmark, Nazanin Mirshahi, Ramon Diaz-Arrastia, Katya Rascovsky, W. Frank Peacock, Timothy E. Van Meter

**Affiliations:** 1 Department of Internal Medicine, Virginia Tech Carilion School of Medicine, Roanoke, VA 24014, USA; 2 Department of Internal Medicine, Edward Via College of Osteopathic Medicine, Blacksburg, VA 24060, USA; 3 Department of Emergency Medicine, Virginia Tech Carilion School of Medicine, Roanoke, VA 24014, USA; 4 Department of Neurology, University of Pennsylvania Perelman School of Medicine, Philadelphia, PA 19104, USAkatyaras@pennmedicine.upenn.edu (K.R.); 5 BRAINBox Solutions, Inc., Richmond, VA 23219, USA; nmirshahi@brainboxinc.com (N.M.); tvanmeter@brainboxinc.com (T.E.V.M.); 6 Department of Emergency Medicine, Baylor College of Medicine, Houston, TX 77030, USA

**Keywords:** traumatic brain injury, cognitive test, mild cognitive impairment, dementia, geriatric

## Abstract

**Background/Objectives:** Older adults are disproportionately affected by traumatic brain injuries (TBIs), representing a significant portion of TBI-related hospitalizations and deaths. The objective of this study was to evaluate the feasibility and effectiveness of BrainCheck (Braincheck, Inc., Austin, TX, USA), a digital cognitive assessment tool, in detecting acute TBI-related cognitive deficits in the context of dementia-related cognitive impairment in older adult emergency department (ED) patients. **Methods:** From March 2020 to November 2023, participants aged 65+ with mild TBI, as defined by the American College of Rehabilitation Medicine (ACRM) diagnostic criteria, and individuals with isolated orthopedic injuries were recruited from 14 U.S. type 1 and 2 trauma centers. After informed consent, each subject was assessed by well-validated neurocognitive tests to characterize pre- and postinjury cognitive status. The Clinical Dementia Rating (CDR) and Functional Activities Questionnaire (FAQ) were used to assess cognitive impairment, with the informant sections used to classify preinjury status. The Rivermead Post-Concussion Symptoms Questionnaire (RPQ16) was used to assess injury-related symptoms, and the tablet-based BrainCheck Battery was tested as a diagnostic platform for injury-related deficits across several functional domains. Spearman’s correlation was used to assess BrainCheck’s internal validity and its relationship with self-reported cognitive symptoms. Technology familiarity was self-reported on a 1 (lowest) to 5 (highest) Likert scale. ROC curves evaluated the tool’s accuracy in identifying cognitive impairment associated with TBI in the context of pre-existing cognitive impairment. **Results:** For the 101 mTBI and 52 orthopedic trauma control patients, BrainCheck demonstrated strong internal validity, with significant correlations among its component tests, indicating its effectiveness in assessing cognitive impairment. However, low correlations with RPQ16 self-reported symptoms suggest that BrainCheck and the self-reported questionnaire assess different aspects of cognitive functions. **Conclusions:** While BrainCheck effectively identified cognitive impairment, the composite battery and scoring did not differentiate TBI and dementia. Technology familiarity did not affect test outcomes. BrainCheck is a useful tool for evaluating cognitive function in adults aged ≥ 65 years with and without TBI in ED settings.

## 1. Introduction

Older adults constitute the largest demographic seeking care for head trauma in the emergency department (ED) [1,2,3,4]. In 2017, individuals aged 65 and older constituted 15.2% of the U.S. population, as reported by the Administration on Aging, yet they accounted for 43.9% of all hospitalizations due to traumatic brain injuries (TBIs) and 38.4% of all TBI-related deaths in the United States [1,5]. Undesirable TBI outcomes trend linearly with age, even beyond 80, and a patient being geriatric (age) is the most significant independent predictor of death or disability from TBI [6,7,8]. Geriatric patients with TBI have the poorest neurological and functional outcomes compared to any other age group, even when normalized for injury severity [9,10,11]. Yet this population remains the most underrepresented population in TBI research, and as a result, there are no objective tools to diagnose or classify geriatric TBI patients accurately.

Geriatric patients are at the highest risk for TBIs in part due to increased fall risk. Individuals over the age of 65 have approximately a 27% likelihood of experiencing a fall each year [9]. This increase is due to many factors, including cardiac episodes such as syncope and commonly co-existing pyramidal, extrapyramidal, and neuromuscular dysfunction [12,13,14]. Low-level falls are the cause of over 80% of geriatric TBIs, and the resulting morbidity increases the risk of subsequent falls [12,15]. Furthermore, TBIs and dementia, which commonly co-exist in older adults, have a well-documented interplay, where TBI exacerbates the likelihood of dementia and vice versa [4,16,17,18,19,20,21].

TBI is increasingly understood to have long-lasting effects, particularly in individuals with pre-existing or pre-symptomatic neurodegenerative diseases [22]. In the geriatric population, TBI often results in incomplete cognitive recovery, a higher likelihood of accelerated cognitive decline over shorter periods, and an elevated risk of developing dementia [4,6,9,11,12,13,14,15,23,24,25,26,27]. The connection between TBI and dementia is notably stronger in cases of multiple previous TBIs [14,27]. Reflecting this evidence, the 2020 Lancet Commission on Dementia recognized TBI as one of twelve modifiable risk factors for dementia [16]. Several biological mechanisms, such as vascular injury, axonal disruption, increased neuroinflammation, and the aggregation of proteins like phosphorylated tau (p-tau) and amyloid beta, may explain these links [17]. In older adults, TBIs can be overlooked or incorrectly diagnosed because their symptoms often resemble those of other conditions prevalent in this age group, such as mild cognitive impairment. This resultant morbidity mandates accurate diagnosis of TBI in this population. Recent studies have also recommended cognitive screening for older adults in the ED following a fall with injury, but tools are needed to implement these recommendations [18,20,21].

Older adults requiring acute care visits after evidence of suspected head trauma commonly receive computed tomography (CT) imaging, as decision guides for CT use in mild TBI (mTBI) patients exclude those over the age of 60 [28,29,30,31,32,33,34]. The diagnosis of mTBI in the ED currently relies on clinical findings. This approach poses challenges in the older adult population due to several factors: the patient history is frequently incomplete, such as missing unwitnessed falls in nursing facilities, compounded by existing memory impairments, and physical signs are non-specific and often obscured by pre-existing neurological impairments [35,36]. Although most geriatric patients undergo head CT imaging, it remains a limited diagnostic and predictive tool for mTBI in this population. TBI-related abnormalities are present on CT in a small percentage of older adult patients evaluated in EDs, yet a significant number of patients have disabling TBI symptoms after negative imaging [31,37,38,39,40]. Consequently, while acute care physicians can use CT imaging to exclude emergencies that require neurosurgical intervention or neurocritical care, they have very little ability to objectively diagnose and risk-stratify acute TBI patients for prognosis [31,41]. Because of these extraordinary challenges in diagnosis and categorization, new tools to aid clinicians in the diagnosis of TBI in older adults are needed. The complex interplay of comorbidities and TBI demands that updating diagnostic tools for geriatric head trauma patients should incorporate objective methods to differentiate existing cognitive impairment or dementia from a new TBI diagnosis. This could be achieved with atrophy measurements from neuroimaging or potentially from biomarkers or physiological data models that help discern profiles specific to this population.

BrainCheck (Braincheck, Inc., Austin, TX, USA) is a computerized cognitive assessment tool that may be used to evaluate cognitive status. It can be accessed via mobile devices like smartphones, tablets, and computers, allowing for both portability and remote testing. BrainCheck provides automated scoring and immediate interpretation, with a brief administration time compared to traditional cognitive screening tools. Despite its short testing duration, it offers comprehensive insights into various aspects of cognitive function. Previously, BrainCheck has been separately validated for identifying concussions and dementia-related cognitive decline [28,35]. In this study our purpose was to evaluate the feasibility and effectiveness of BrainCheck in distinguishing age-normal cognitive performance versus cognitive impairment, in the geriatric population presenting to the ED with suspected head injuries.

## 2. Methods

We performed an observational cohort study using male and female patients drawn from two parallel clinical studies that shared standardized protocols and study infrastructure. The study was registered with ClinicalTrials.gov (NCT04423198) on 8 June 2020. All participating EDs received centralized IRB approval. Two groups of trauma subjects, non-head trauma (trauma controls) and head trauma with mTBI (TBI arm), were recruited from the EDs of 12 level I trauma centers and 2 level II trauma centers in the United States. After informed consent, adults aged ≥ 65 years who met the American College of Rehabilitation Medicine (ACRM) mTBI criteria were enrolled and evaluated as a target condition in a pilot study of geriatric trauma subjects, alongside non-head-injury trauma subjects [42].

All patients received standard-of-care trauma evaluations and were administered the BrainCheck Battery neurocognitive test. Patients taking BrainCheck neurocognitive testing were asked to grade their familiarity with touchscreen and tablet use, using a Likert scale of 1–5, where 5 is most familiar, and 1 was least familiar. The first cohort of 84 patients (*n* = 51 mTBI and *n* = 33 trauma controls) was from the HeadSMART Geriatric Feasibility Study, which enrolled from March 2023 to November 2023.

The second cohort was obtained from the ongoing HeadSMART II Study, the methods of which have been previously reported, with patients enrolled between March 2020 and July 2023 [29]. This sample of patients represented an adult blunt-head-injury cohort, with all subjects adjudicated as having a mild TBI diagnosis by an independent panel of experts using the ACRM criteria, neuroimaging, and expert clinical assessment [29]. A STROBE checklist is provided as a supplement (Table S3).

### 2.1. Neurocognitive Testing Procedures

The HeadSMART Geriatric subjects were evaluated by standardized assessment instruments for determining cognitive status and dementia that are in routine clinical use in neurology, rehabilitation, and ED environments. These subjects represent the intended use population and an appropriate trauma control population (non-head trauma controls, TC). Pen and paper and digital neurocognitive testing procedures were performed on subjects by trained research staff during the acute ED visit. Standardized questionnaires included the Clinical Dementia Rating Scale (CDR) [43,44], Functional Activities Questionnaire (FAQ) [45,46,47], and Rivermead Post-Concussion Questionnaire-16 question (RPQ16) [48,49,50], which were collected to further characterize the subjects. In each case, the validated standardized thresholds were used to characterize the status of the subjects. Preinjury cognitive status (normal versus impaired) was assessed using the informant section of the CDR and FAQ, wherein the caregiver questionnaires are used to establish preinjury cognitive impairment or dementia stage. These assessments were compared with the effects of mTBI, as seen by BrainCheck neurocognitive tests and MoCA as a gold-standard comparator.

### 2.2. BrainCheck Digital Neurocognitive Assessment

The BrainCheck Battery was administered on a dedicated tablet device, preloaded with BrainCheck software (version 5.8.3.FAB20133F, Austin, TX, USA) [35]. The research staff explained the testing procedure to the subject, who then self-administered the Battery, advancing through each individual software portion, as prompted in screen by the software. The BrainCheck software includes a weighted algorithm known as the Clinical Score, which is a weighted composite of all individual tests (Digit Symbol, Trails A and B, Immediate and Delayed Recall, Stroop, Flanker, Coordination), and has an algorithm for detecting malingering. The normative database used for age adjustment is also a part of the software database and standardized score results.

### 2.3. Statistical Analysis

Comparison of patient demographics was performed by Spearman’s correlation coefficient, and distributions of scores from BrainCheck composite and individual test scores were compared between groups by Wilcoxon tests to determine differences in medians. All plots, graphics, and statistical comparisons were computed in R (version 4.4.2).

## 3. Results

**Figure 1:**
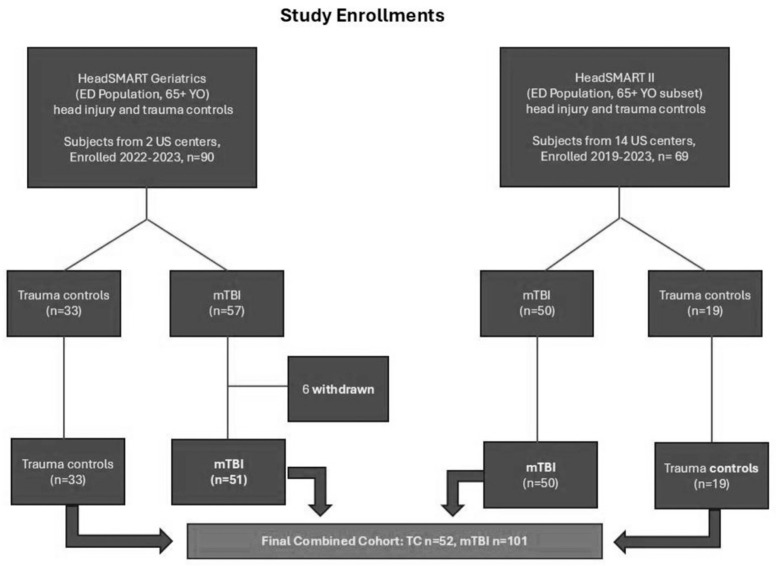
STROBE Chart of the Included Study Participants.

A total of 153 subjects were included in this analysis. All were 65 years of age and older, with either TBI (ACRM mTBI positive; *n* = 101) or were enrolled as orthopedic trauma controls, defined as having isolated orthopedic trauma (Figure 1).

### 3.1. Demographic Characterization

The non-head-injury trauma controls were compared with trauma from head injuries (TBI group) to assess demographic differences (Table 1). TBI patients were slightly older than trauma controls (73 vs. 70 years, *p* = 0.041), although the trauma control cohort range fell within that of the TBI patients. There were similar numbers of females (46% vs. 56%, *p* = 0.24) in both cohorts. TBI patients presented to the ED more than 2 h earlier than trauma controls (median 1.8 vs. 4.1 h, *p* = 0.005) and had their first study blood draw >10 h earlier (median 10.3 vs. 20.7 h, *p* = 0.019), yet median discharge times were shorter for the TBI cohort (median 32.2 vs. 71.1 h, *p* = 0.05). Differences were seen in injury mechanism, with falls being the majority of all cases, which were fewer in trauma controls.

Prior diagnoses of comorbidities were similar between groups. Table 2 characterizes the intake symptoms in the TBI group. Approximately half of the subjects had isolated head trauma, while the other half had additional bodily injuries. Most subjects had a GCS of 15 (96%), and 17.8% were found to have a positive head CT. The most prevalent clinical sign of TBI was loss of consciousness (44.6%), followed by disorientation or confusion (38.6%), focal neurological deficit (31.7%), and post-traumatic amnesia (21.8%). A total of 39 of 99 subjects with available data (38%) were discharged with a diagnosis of TBI.

### 3.2. Digital Neurocognitive Assessments for TBI in Geriatric Trauma Patients

To understand whether questionnaires with self-reported information about cognitive symptoms were aligned with visual tablet-based digital neurocognitive testing metrics, Spearman’s correlation coefficients were calculated comparing the RPQ16 cognitive questions reporting poor concentration, forgetfulness and memory issues, and taking longer to think. The correlation matrices are included in Supplemental Table S1.

With the RPQ16 questions, some degree of internal consistency was found in testing TBI subjects with normal preinjury status, with a moderate positive correlation between poor concentration and forgetfulness (ρ = 0.61, *p* < 0.001) and taking longer to think (ρ = 0.46, *p* < 0.001), and between forgetfulness and taking longer to think (ρ = 0.44, *p* < 0.001). When these self-reported questions were correlated to BrainCheck Battery tests, weak correlations were seen between ‘taking longer to think’ and Immediate (ρ = 0.26, *p* = 0.02) or Delayed Recall (ρ = 0.29, *p* < 0.001), as was a weak negative correlation between the Flanker score and forgetfulness (ρ = −0.23, *p* = 0.042). In contrast, the individual tests within the BrainCheck digital (gamified) format showed stronger correlations with one another, suggesting digital cognitive testing has internal validity. For example, Immediate and Delayed Recall show positive correlation (ρ = 0.47, *p* < 0.001), as do Trails A and B (ρ = 0.51, *p* < 0.001), as expected for highly similar tests for the same cognitive domains. Other significant, but less predictable, correlations across different cognitive domains within the BrainCheck Battery included Trails B and Digit Symbol Substitution (ρ = 0.54, *p* < 0.001) or Stroop Test and Balance (ρ = 0.48, *p* < 0.001). The overall Clinical Score composite correlated with all individual component tests, with the strongest correlations for Digit Symbol (ρ = 0.75, *p* < 0.001) and Trails A (ρ = 0.74, *p* < 0.001), followed by Immediate and Delayed Recall (ρ = 0.59 and 0.56, respectively, *p* < 0.001). This data supports the internal validity of the BrainCheck Battery when used in the population of geriatric mTBI and trauma patients. Correlations between digital neurocognitive tests and RPQ16 cognitive questions are included for TBI and TC in supplemental data tables (see Table S1 and S2).

### 3.3. Effect of Preinjury Impairment on Individual Neurocognitive Tests

**Figure 2:**
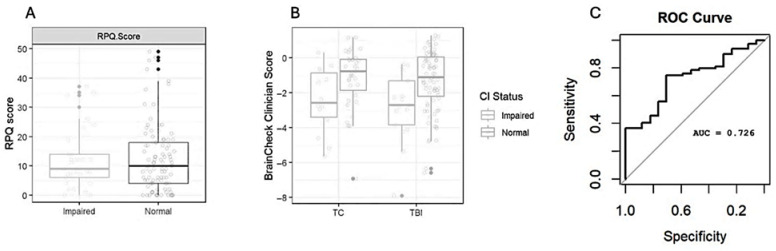
**BrainCheck detection of impaired status in non-head and head trauma subjects.** (**A**). Symptom assessment by Rivermead (RPQ) in subjects of differing preinjury cognitive impairment status. (**B**). BrainCheck composite Clinician Score in clinical subgroups, separated by preinjury cognitive status. (**C**). Receiver Operator Characteristic (ROC) Curve for distinguishing CI status = preinjury cognitive status by informant assessments. Abbreviations: RPQ, Rivermead Post-Concussion Questionnaire-16 question; TC, trauma control; TBI, mild traumatic brain injury; CI, cognitive impairment; AUC, area under the ROC curve.

The CDR and FAQ each have informant sections that were used to evaluate prior-history cognitive status for each subject in HeadSMART Geriatric subjects and approximate trauma patients’ preinjury cognitive impairment or dementia stage. Separating subjects by CDR/FAQ-defined cognitive status and by injury type demonstrated that preinjury status was associated with lower BrainCheck scores in both groups (Figure 2). Figure 2A shows that total RPQ16 scores do not discriminate between patient groups at the acute evaluation. Figure 2B shows the corresponding composite scores for the BrainCheck Battery (Clinician Score). Irrespective of injury type, cognitive deficits are identified, as indicated by the Clinician Score distributions. Looking within TBI subjects alone, BrainCheck testing was used to predict conversion from preinjury normal cognitive status to TBI-related cognitive functional deficits, which correlated with MoCA measurements at the same time in the ED. The overall TBI prediction for all subjects with BrainCheck was found to have an AUC of 0.73 (Figure 2C). The transition after head injury to acute cognitive impairment was reflected in the MoCA scores, which correlated with BrainCheck scores (r = 0.68). The concordance of the BrainCheck Clinician Score and MoCA was similar in TC, as reported previously in neurology patients [28]. Preinjury status was also maintained in the subjects with preinjury cognitive impairment, as indicated by comparing the CDR/FAQ informant section status and MoCA (0 trauma controls converted to CI by non-CNS injury. Exploration of individual component metrics from digital testing (e.g., Trails B or Digit Symbol) may be able to further discriminate differences between acute-brain injury-related cognitive deficits and chronic neurodegeneration.

### 3.4. Technology Familiarity as a Factor Affecting Tablet-Based Neurocognitive Testing

**Figure 3:**
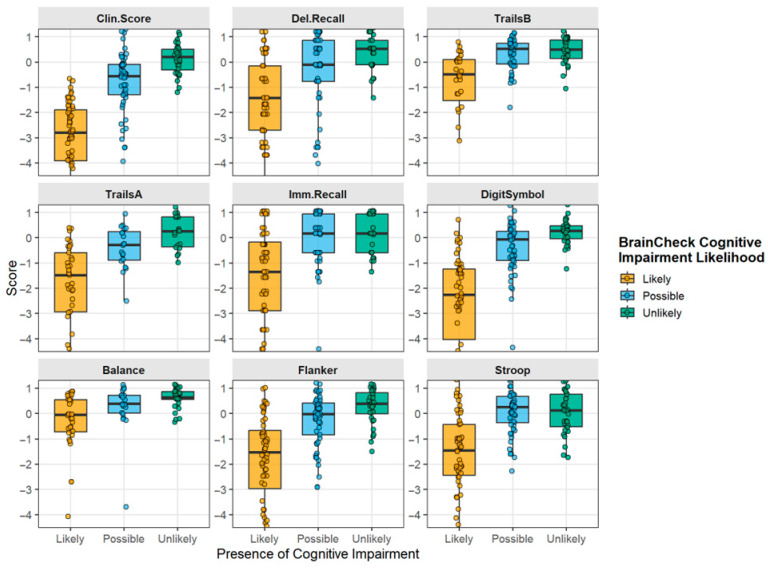
**Relationship of individual subtest scores to BrainCheck cognitive impairment likelihood calls in the geriatric TBI subjects.** Box plot distributions for the BrainCheck composite Clinician Score, calculated by a weighted algorithm, and for each of the individual assessments in the digital battery. Trauma control (TC) and TBI groups are plotted with subsets shown by preinjury cognitive impairment (CI Status), as determined by the informant sections of the CDR and FAQ. Clin.Score, BrainCheck calculated composite “Clinician Score”; Del.Recall, Delayed Recall; Imm.Recall, Immediate Recall; open dots, data points from individual patients; closed dots, outliers; solid line, median.

**Figure 4:**
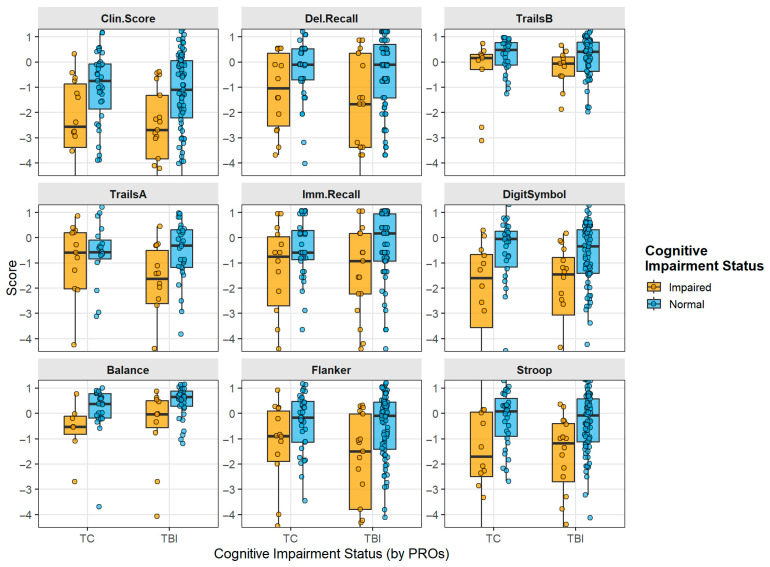
**Analysis of BrainCheck cognitive tests and preinjury cognitive impairment status.** Box plot distributions for the BrainCheck composite Clinician Score, calculated by a weighted algorithm, and for each of the individual assessments in the digital battery. Trauma control (TC) and TBI groups are plotted with subsets shown by preinjury cognitive impairment (CI Status), as determined by the informant sections of the CDR and FAQ. Clin.Score, BrainCheck calculated composite “Clinician Score”; Del.Recall, Delayed Recall; Imm.Recall, Immediate Recall; open dots, data points from individual patients; closed dots, outliers; solid line, median.

**Figure 5:**
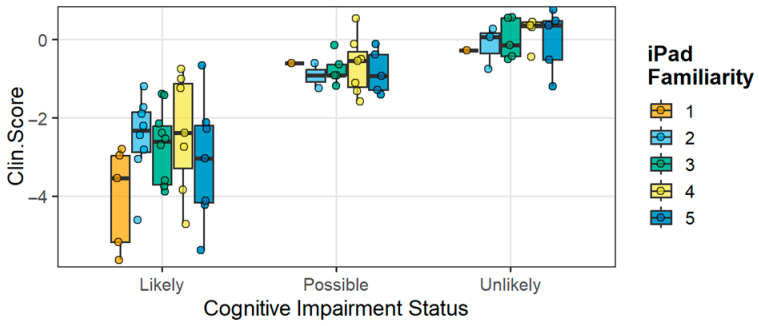
**Effect of familiarity of iPAD use on the composite BrainCheck Clinical Score and Call.** Distributions of BrainCheck Clinician Score separated by self-reported familiarity with touch pad phone or tablet technology. Plotted left to right from Likert score of 1 (left) to 5 (right) for least to most familiar. Clin.Score, BrainCheck calculated composite “Clinician Score”; open dots, data points from individual patients; closed dots, outliers; solid line, median; dotted line, suggested threshold for impairment.

Figure 3 shows the results of the overall composite scores from BrainCheck testing, and for each component test for TBI subjects, separated by the overall categorization of the software as Likely, Possible, or Unlikely to be cognitively impaired. Analysis of BrainCheck scores within the preinjury cognitive status subgroups of both TBI and trauma controls is shown in Figure 4. Median scores were stable within cognitive impairment groups, with the preinjury CDR- and FAQ-defined preinjury impairment status showing the strongest relationship to BrainCheck Scores. When subject scores were characterized with respect to technology familiarity, no major differences were observed across scores defined by BrainCheck Clinician Score, though these composite scores showed clear differences in impairment between groups (Figure 5). This unexpected result indicated that digital neurocognitive testing was not affected by the subject’s reported technological familiarity level and was feasible and effective in the geriatric trauma population in the ED environment.

## 4. Discussion

Consistent with previous research, BrainCheck demonstrated an ability to measure cognitive impairment [28,30]. The BrainCheck Clinical Score effectively distinguishes individuals with pre-existing cognitive impairment from those without. Pearson and Spearman correlations confirmed the internal consistency of both the BrainCheck program and its individual tests. Tests like Trails A, Trails B, and Digit Symbol, which assess similar cognitive processes, showed moderate to high correlations. Similarly, in the non-head-injured subjects who were cognitively impaired preinjury, MoCA was positively correlated with the “Likely” CI BrainCheck clinician scores, as previously reported [28]. This alignment with MoCA was also seen in TBI-related changes in subjects that were preinjury normal (CDR/FAQ) and shifted to impaired status (MoCA, BrainCheck). Standard neuropsychological tests, which measure specific cognitive skills, typically correlate well with other tests targeting the same processes. For example, Immediate and Delayed Recall both assess verbal memory and usually exhibit high correlations [37]. Similarly, BrainCheck demonstrates high correlations between tests evaluating similar cognitive functions, suggesting that its tablet-based versions are valid in assessing their intended domains.

When evaluating cognitive symptoms in geriatric patients with mTBI and trauma, it is essential to understand the validity and applicability of assessment tools. The RPQ16 questionnaire and BrainCheck Battery both show strong internal validity, as evidenced by consistent correlations within their respective measures, with the RPQ16 displaying moderate correlations among self-reported cognitive symptoms and the BrainCheck Battery demonstrating robust test correlations. However, the weak correlations between RPQ16 symptoms and BrainCheck metrics highlight a lack of external validity between them, suggesting they assess cognitive functions differently. Thus, these tools should be viewed as complementary rather than interchangeable for evaluating cognitive symptoms in geriatric mild TBI and trauma patients.

While BrainCheck appears equipped to detect cognitive impairment, its ability to differentiate between dementia and TBI remains unclear. The data indicated that BrainCheck was less effective in distinguishing between the preinjury cognitive impairment in the trauma controls or TBI and any postinjury TBI-induced impairment (Figure 2 and Figure 4). The heterogeneity of dementia and of TBI may necessitate a more robust characterization of participants and a larger study to detect significant differences among varying cognitive presentations. In the future, integrating blood-based biomarkers with cognitive testing could enhance the differentiation between dementia and mTBI. Biofluid biomarkers may provide additional data that could lead to more accurate diagnoses when combined with cognitive assessments. Further research is needed to explore the potential of biomarkers in conjunction with cognitive testing tools, such as BrainCheck, to improve diagnostic precision and treatment strategies for these conditions.

Some individual assessments were more effective at differentiating cognitive impairment between cognitively normal and cognitively impaired groups than others, which may also account for the limited differentiation between dementia and TBI. Based on these data, Trails A, Flanker, and Digit Symbol attributed more strongly to the overall BrainCheck Clinical Score, while Balance seemed to account for less of the variance. Digit Symbol testing showed good separation in scores, supporting a significant difference between Likely, Possible, and Unlikely cognitive impairment “calls”. Previous research found inconsistencies when it came to Digit Symbol’s ability to show significant differences between cognitively healthy and cognitively impaired individuals [28]. Digit Symbol exhibited a larger standard deviation within both the likely impaired group, indicating a wider spread of scores, producing additional data regarding Digit Symbol’s inconsistencies. When comparing impaired individuals to those with normal cognitive function, the Trails A, Delayed Recall, Flanker, and BrainCheck Stroop tests demonstrated greater effectiveness in detecting Likely cognitive impairment. Conducting additional research with larger sample sizes could help reduce this variance and tighten standard deviations, thereby increasing the chances of achieving statistical significance for comparisons.

Participants in the study were asked to rate their familiarity with using a tablet device. The level of familiarity reported by participants did not correlate with the likelihood of cognitive impairment, whether categorized as Likely, Possible, or Unlikely. This suggests that the participants’ self-reported comfort and experience with tablet use did not influence their ability to complete the cognitive assessments conducted on these devices. The use of a tablet-based cognitive measure had minimal impact on the effectiveness of the assessment in detecting cognitive impairment or the assessment’s diagnostic accuracy. There has been a concern among researchers and clinicians that older adults would find digital and tablet-based assessment measures daunting or confusing, thereby limiting their efficacy [38,41]. Another concern is that mild or more severe cognitive impairments may reduce the efficacy of tablet-based assessments [51,52]. The current data suggests these concerns are unfounded, following the trend in research that supports computerized cognitive tests as effective tools in older adults [53]. The utility of digital health devices and digital data innovation in characterizing clinical neurology populations has been advancing steadily, particularly in the past decade [54]. The use of machine learning and artificial intelligence for learning data profiles and applying these to diagnostic and prognostic signatures is a major area of research and development in recent years. Therefore, having well-annotated clinical data sets in specific disease types will allow digital metrics to be combined with other biomarker types, such as neuroimaging and biofluid biomarkers, to better indicate and monitor disease [55,56]. This feasibility study suggests that cognitive deficits can be detected in brief digital interfaces (tests) that can fit within the clinical workflows of the acute care setting. Such approaches have already been in development in neurology clinics, paving the way for more widespread use in precision neurology models [56].

### Strengths and Limitations

Using the BrainCheck tool to evaluate cognitive function in the geriatric population provided valuable insights into the feasibility of digital cognitive assessments in the ED. One limitation of this study is the creation of the dataset by the combination of two separate trials. Thus, while our findings are robust, this strategy serves as a generalizability limitation when considering their application to other populations. Second, the reliance on caregiver reports to determine baseline cognitive status may contribute to the difficulty in distinguishing between TBI and dementia. While caregiver insights can be valuable, they may introduce subjective bias or inaccuracies due to varying levels of understanding and observation skills regarding cognitive changes. To address this, future research should consider incorporating a broader range of objective assessment tools and methodologies to establish baseline cognitive function more reliably. Additionally, integrating biomarker analysis could enhance the ability to differentiate between cognitive impairments caused by TBI and those due to dementia, providing a more comprehensive understanding of the underlying conditions. Another limitation is the relatively small and potentially homogeneous sample size, which may not fully capture the diverse presentations of cognitive impairments in the geriatric population. Expanding the sample size and diversifying participant demographics would help ensure that findings are more generalizable and representative of the broader population. Finally, the study’s cross-sectional design limits the ability to draw conclusions about the long-term impacts of TBI and dementia on cognitive function, underscoring the need for longitudinal studies to track changes over time.

## 5. Conclusions

In conclusion, this study underscores the potential of BrainCheck as a valuable and effective cognitive assessment tool, particularly within ED settings. Its brevity, portability, and ease of use enhance opportunities for evaluating cognitive function in acute care environments, offering critical insights that can aid emergency medicine professionals in making more informed diagnostic decisions. By demonstrating the feasibility and utility of using BrainCheck, this research highlights its role in improving the quality of care for older adults facing traumatic brain injuries and cognitive impairments. Future research may address the value of combining BrainCheck with biofluid biomarker tests to enhance discrimination between older adult patients with cognitive impairment and those with cognitive impairment and TBI, thus ultimately enhancing the accuracy of its ED diagnostic capabilities.

## Figures and Tables

**Table 1:** Demographic information for geriatric trauma subjects.

	TC	TBI	*p*-Value
	(*n* = 52)	(*n* = 101)	
Age	70.0 (65.8–75.0)	73.0 (68.0–78.0)	0.041
Sex			0.24
Female	24 (46.2%)	57 (56.4%)	
Male	28 (53.8%)	44 (43.6%)	
Race			0.26
White	37 (71.2%)	81 (80.2%)	
African American/Black	15 (28.8%)	17 (16.8%)	
Asian	0 (0.0%)	1 (1.0%)	
Not Reported	0 (0.0%)	2 (2.0%)	
Education			0.92
Less than a high school education	3 (5.8%)	8 (7.9%)	
High school degree or equivalent (e.g., GED)	11 (21.2%)	23 (22.8%)	
Some college	28 (53.8%)	54 (53.4%)	
Graduate or professional degree	8 (15.3%)	15 (14.9%)	
Missing	2 (3.8%)	1 (1.0%)	
Vital signs and clinical measures			
Heart rate (beats per minute) at intake			0.35
Median (IQR)	77.0 (72.0–86.5)	76.0 (68.0–84.0)	
Missing	1 (1.9%)	0 (0%)	
Systolic BP (mmHg.) at intake			0.55
Median (IQR)	152.0 (141.0–168.5)	150.0 (133.0–167.0)	
Missing	1 (1.9%)	0 (0%)	
Diastolic BP (mmHg.) at intake			0.65
Median (IQR)	79.0 (71.0–86.5)	78.0 (71.0–84.0)	
Missing	1 (1.9%)	0 (0%)	
Body Mass Index at intake	26.0 (22.5–30.6)	27.3 (23.9–30.8)	0.38
ED Evaluation time from injury (h)	4.1 (1.7–22.6)	1.8 (1.0–6.3)	0.005
BC Duration (min)			0.68
Median (IQR)	16.0 (13.0–19.0)	15.0 (13.0–19.5)	
Missing	2 (3.8%)	2 (2.0%)	
Mode of arrival			0.28
Ambulance	23 (44.2%)	55 (54.5%)	
Friend/family member	9 (17.3%)	21 (20.8%)	
Self-transport	8 (15.4%)	12 (11.9%)	
Transfer from other facility	12 (23.1%)	12 (11.9%)	
Missing	0 (0.0%)	1 (1.0%)	
Mechanism of injury			0.01
Fall from standing	32 (61.5%)	77 (76.2%)	
Motor Vehicle Collision (not ejected)	6 (11.5%)	7 (6.9%)	
Head struck by/against object	0 (0.0%)	7 (6.9%)	
Pedestrian struck by vehicle	1 (1.9%)	2 (2.0%)	
Pedal Cycle (non-motorized with helmet)	1 (1.9%)	1 (1.0%)	
Other	12 (23%)	6 (6%)	
Missing	1 (1.9%)	1 (1.0%)	

**Table 2:** Injury intake characteristics of study participants.

TBI-Related Injury Characteristics	
	TBI
	(*n* = 101)
Type of injury	
Blunt Head Trauma	53 (52.5%)
Blunt Head Trauma with Additional Trauma Injury	48 (47.5%)
Head Neuroimaging results (CT/MRI)	
Negative	79 (78.2%)
Positive	18 (17.8%)
No CT	4 (4.0%)
GCS	
14	4 (4.0%)
15	97 (96.0%)
ACRM	
ACRM−	24 (23.8%)
ACRM+	77 (76.2%)
Headache at intake	
Yes	68 (67.3%)
No	33 (32.7%)
Not applicable	0 (0.0%)
Severe headache	
Yes	26 (25.7%)
No	41 (40.6%)
Not applicable	0 (0.0%)
Unknown	14 (13.9%)
Missing	20 (19.8%)
Loss of consciousness	
Yes	45 (44.6%)
Not Sure	9 (8.9%)
No	47 (46.5%)
Not applicable	0 (0.0%)
Post-traumatic amnesia (PTA)	
Yes	22 (21.8%)
Not Sure	1 (1.0%)
No	78 (77.2%)
Not applicable	0 (0.0%)
PTA (Affecting memories before injury)	
Yes	14 (13.9%)
No	8 (7.9%)
Not applicable	79 (78.2%)
PTA (Affecting memories after injury)	
Yes	17 (16.8%)
No	5 (5.0%)
Not applicable	79 (78.2%)
Disorientation/Confusion	
Yes	39 (38.6%)
Not Sure	2 (2.0%)
No	60 (59.4%)
Not applicable	0 (0.0%)
Focal neurological deficit	
Yes	32 (31.7%)
Not Sure	3 (3.0%)
No	66 (65.3%)
Not applicable	0 (0.0%)
Post-traumatic seizure	
Yes	0 (0.0%)
Not Sure	2 (2.0%)
No	99 (98.0%)
Not applicable	0 (0.0%)

ACRM—American College of Rehabilitation Medicine guideline.

## Data Availability

The data generated and analyzed during this study are not publicly available but are available from the corresponding author upon reasonable request.

## Supplementary Materials

The following supporting information can be downloaded at: https://www.mdpi.com/article/10.3390/diagnostics16030400/s1, Table S1: Correlation of Cognitive Questions in RPQ16 with digital neurocognitive tests; Table S2: Correlations of digital neurocognitive test scores in TBI and controls; Table S3: STROBE Statement—checklist of items that should be included in reports of observational studies.
