# Supplementary figures and images for: The Use of Digital Neurocognitive Assessments to Assess Traumatic Brain Injury and Dementia in Older Trauma Patients: An Emergency Department Feasibility Study
Source: Diagnostics (Basel). 2026 Jan 27;16(3):400. doi: 10.3390/diagnostics16030400 (PMC12897280; doi:10.3390/diagnostics16030400)

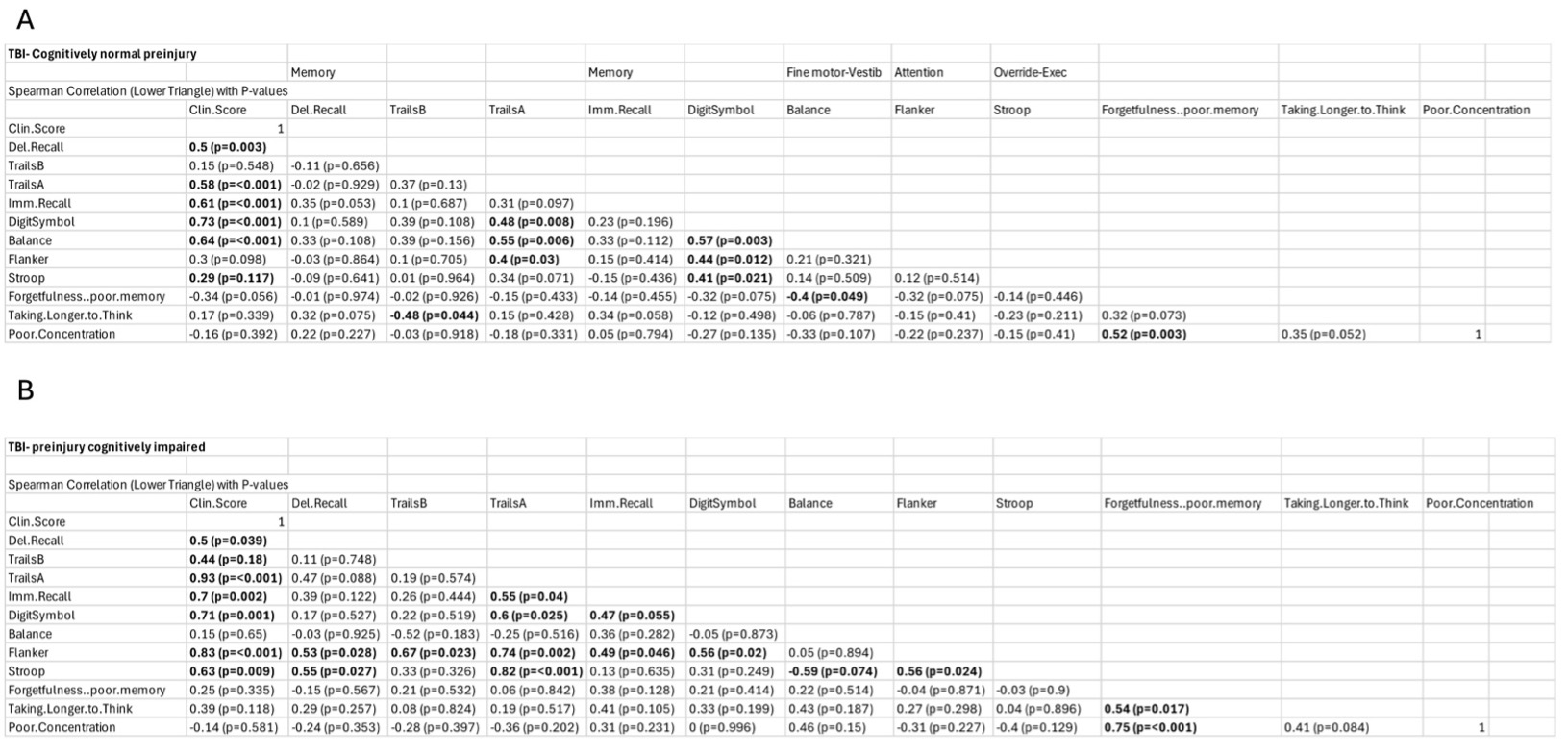

Supplement: Supplementary file 1 [file diagnostics-16-00400-s001.zip › Supplemental Table S1.tiff]

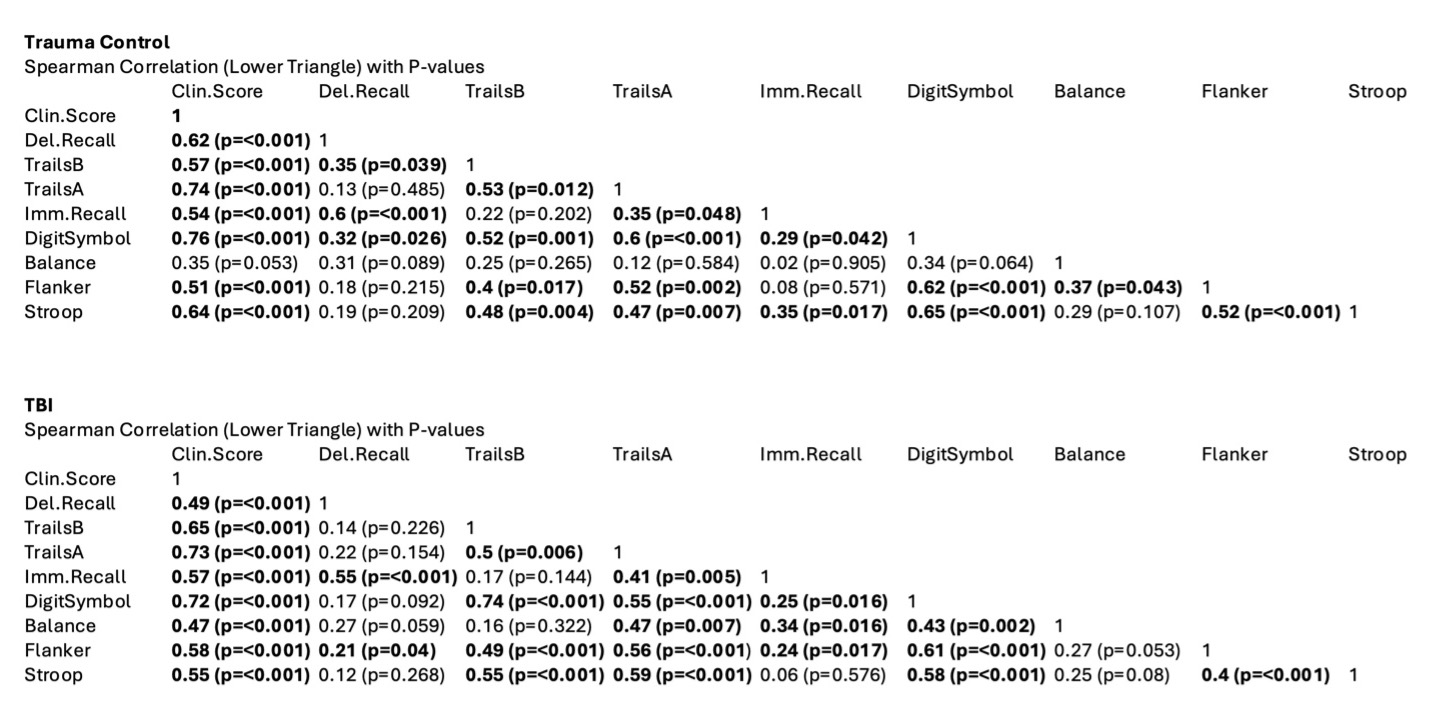

Supplement: Supplementary file 1 [file diagnostics-16-00400-s001.zip › Supplemental Table S2.tiff]
